# Collecting the evidence: mechanistic insights into *Akkermansia muciniphila’s* impact on aging and systemic inflammation

**DOI:** 10.3389/fimmu.2025.1733575

**Published:** 2026-01-21

**Authors:** Aleksandr I. Vorontsov, Andrey A. Kruglov, Ekaterina O. Gubernatorova

**Affiliations:** 1 Engelhardt Institute of Molecular Biology, Russian Academy of Sciences, Moscow, Russia; 2 Faculty of Biology and Belozersky Institute of Physico-Chemical Biology, Lomonosov Moscow State University, Moscow, Russia; 3 Deutsches Rheuma-Forschungszentrum (DRFZ), An Institute of the Leibniz Association, Berlin, Germany; 4 Center for Precision Genetic Technologies for Medicine, Engelhardt Institute of Molecular Biology, Russian Academy of Sciences, Moscow, Russia

**Keywords:** *Akkermansia muciniphila*, healthy aging, hematopoiesis, inflammaging, intestinal barrier, metabolism, probiotics

## Abstract

*Akkermansia muciniphila* is a Gram-negative, mucin-degrading anaerobic bacterium that constitutes an important component of the human commensal microbiota. A reduction in its abundance is associated not only with intestinal barrier dysfunction but also with systemic inflammation and age-related metabolic disorders. Given its distinctive biological properties, *A. muciniphila*-based probiotics emerged as a promising strategy for alleviating age-associated metabolic and hematopoietic decline. Nonetheless, current experimental evidence is somewhat inconsistent. Accumulating data indicate that *A. muciniphila* can exert both beneficial and deleterious effects on systemic inflammation and tissue homeostasis, with outcomes strongly influenced by bacterial dose, host status, and the surrounding microbial and dietary context. While several studies report that *A. muciniphila* supplementation reinforces mucosal barrier integrity and mitigates chronic inflammation, thereby preserving bone marrow homeostasis; others describe deleterious effects, including mucus layer erosion and heightened metabolic endotoxemia. In this review, we summarize these findings and propose mechanistic explanations for how *A. muciniphila* may benefit the aging process, ultimately contributing to improved health and quality of life in the elderly population. Additionally, we identify key gaps in current knowledge and outline priorities for future mechanistic and longitudinal human studies needed to define when and how *A. muciniphila*-based interventions can be used safely and effectively during aging.

## Introduction

Aging is a natural biological process characterized by a progressive decline in organ function, primarily driven by the diminished ability of cells to proliferate and respond to physiological stress. At the systemic level, these age-related changes disrupt homeostatic equilibrium, foster chronic inflammatory and degenerative conditions, and increase disease susceptibility due to impaired immune competence (1). Beyond intrinsic cellular deterioration, the intestinal microbiota also undergoes substantial compositional and functional shifts with age, which further contribute to the onset of age-associated disorders, including inflammatory bowel disease, systemic inflammation, and cancer (2).

The interplay between the intestinal microbiota and the host’s lifespan has emerged as one of the focuses in aging research. Mounting evidence underscores that preserving high microbial diversity is essential for healthy aging, as the relative abundance of beneficial commensals typically diminishes with age, accompanied by the expansion of opportunistic taxa (3). This age-related dysbiosis compromises intestinal barrier integrity by thinning the mucus layer and impairing epithelial cell renewal, thereby facilitating microbial products translocation into the underlying tissues. Such breaches in barrier function trigger both local and systemic inflammatory cascades (4). The physiological relevance of these compositional alterations was demonstrated in murine models, where fecal microbiota transfer from aged donors to young recipients significantly increased intestinal permeability and elevated circulating inflammatory cytokines, particularly IL-6 and TNF. In line with this, transplanting microbiota from young to old recipients can reverse the detrimental effects of age-related dysbiosis (5).

Interventions designed to restore a more “youthful” microbiota composition may partially counteract the physiological and metabolic alterations associated with aging and dysbiosis. Comparative analyses of gut microbial communities in young mice, centenarians, and healthy older adults identified specific taxa correlated with healthy aging, including *Akkermansia muciniphila* from the phylum *Verrucomicrobiota*. Several studies consistently demonstrated that *A. muciniphila* is more abundant in young adults and centenarians (6) than in elderly individuals with chronic disease (7). These observations suggest that high levels of *A. muciniphila* may serve as a prognostic biomarker indicative of increased healthy lifespan and preserved physiological function during aging.

## The role of *A. muciniphila* in the regulation of intestinal inflammation and cancer

Aging is accompanied by a chronic, low-grade inflammatory state termed “inflammaging” and has been extensively summarized in the literature (8, 9). It is driven by cellular senescence and its associated secretory phenotype, mitochondrial dysfunction, immune dysregulation, and sustained production of proinflammatory mediators. Age-related impairment of the intestinal barrier and microbial dysbiosis contribute additional sources of systemic inflammation through the translocation of microbial metabolites and toxins, which can also induce mutagenic events that alter the proliferative dynamics of intestinal epithelial cells. Collectively, these processes establish a tumor-promoting microenvironment in the elderly, fostering tumor cell survival and expansion, as well as genomic instability, angiogenesis, and immune evasion. Emerging evidence highlights the dual role of the microbiome in cancer, as specific microbial taxa can either promote or suppress tumor growth (9). For instance, genotoxic *Escherichia coli* was shown to directly induce oncogenic mutations, particularly within the *APC* gene, thereby contributing to colorectal carcinogenesis (10). Similarly, *Fusobacterium nucleatum* can persist within tumor tissues and facilitate immune evasion by suppressing T cell–mediated antitumor responses, while also enhancing chemoresistance through the activation of autophagy pathways (11). In contrast, enrichment of *Akkermansia muciniphila* has been consistently associated with favorable clinical outcomes and is considered a prognostic indicator of improved response and survival in malignant disease (12).

*Akkermansia muciniphila* a is a key beneficial intestinal symbiont that has recently been considered a next-generation probiotic due to its inflammation protective and immunomodulatory properties (13). A distinctive feature of this bacterium is its ability to enzymatically cleave intestinal mucin glycoproteins and use their hydrolysis products as the sole source of carbon and nitrogen. This results in renewal and thickening of the mucin layer, improved intestinal barrier function, and reduced inflammation (14, 15). *A. muciniphila* also synthesizes Amuc_1100 protein on its surface (16). This protein plays a key role in colonization, but also increases the expression of tight junction proteins by intestinal epithelial cells, such as occludin and claudin. Another secreted protein, Amuc_1409, improves barrier function by increasing the proliferation and regeneration of intestinal stem cells in *ex vivo* and *in vivo* models of naturally aged mice (17). Thus, it has been established that *A. muciniphila* can improve the integrity of the intestinal barrier and reduce the penetration of pathogens and their components into the deep tissues.

Reduction of intestinal inflammation mediated by *Akkermansia muciniphila* is considered the principal mechanism underlying its beneficial effects in the elderly, as mucosal inflammation has been implicated in the pathogenesis of malignancies such as colorectal (18) and prostate cancer (19). Nonetheless, the literature presents contradicting evidence regarding the bacterium and its derivatives in various inflammatory disease models. On the one hand, administration of low doses of *A. muciniphila* in dextran sulfate sodium (DSS)-induced colitis was shown to attenuate clinical symptoms, reduce inflammatory cytokine levels, and enhance mucus production (20, 21). Similarly, in the azoxymethane/DSS (AOM/DSS) model of colorectal cancer, *A. muciniphila* exerted a protective effect through the activation of cytotoxic lymphocytes (19), while in the Apc^Min/+^ model, tumor burden was reduced via enhanced activity of antitumor macrophages (22). Human studies further corroborate these findings, demonstrating that the presence of *A. muciniphila* in the gut microbiome correlates with improved therapeutic efficacy of both targeted immunotherapies (23) and immune checkpoint inhibitors (24). It should be noted that these observations are largely based on associative analyses in relatively small and selected patient cohorts, and they do not yet establish a direct causal role for *A. muciniphila* in mediating therapeutic response. On the other hand, several reports describe potential adverse consequences of *A. muciniphila* overabundance in model systems. Excessive colonization can disrupt the equilibrium between mucin synthesis and degradation, leading to mucus layer thinning and compromise of the intestinal barrier (25, 26). For example, administration of high bacterial doses in an *in situ* colorectal cancer model exacerbated colitis and accelerated tumor progression (27). Therefore, controlled modulation of the microbiota through *A. muciniphila* supplementation may represent a promising adjunctive approach for the prevention and management of intestinal inflammation and colorectal cancer, provided that dosage, form of delivery and host context are carefully optimized. Taken together, these data suggest that *A. muciniphila* shapes the inflammatory-tumor axis through a balance of barrier-protective and potentially barrier-disruptive activities. By reinforcing the mucus layer, modulating immune cell effector functions, and influencing microbial metabolites, *A. muciniphila* may constrain inflammatory carcinogenesis under homeostatic conditions, yet under barrier-compromised or fiber-deprived states its mucin-degrading capacity could instead amplify epithelial stress and oncogenic signaling. Thus, in the context of cancer, *A. muciniphila* should not be viewed as uniformly protective or harmful, but rather as a context-dependent modulator whose net impact is determined by the broader inflammatory and microbial milieu. The apparent discrepancies between studies reporting beneficial versus deleterious effects of *A. muciniphila* likely arise from differences in several key variables (28). These include bacterial dose and duration of exposure, the use of viable versus pasteurized preparations, host age and baseline metabolic or inflammatory status, dietary fiber content, and the composition of the co-resident microbiota that shapes cross-feeding and competitive interactions. In addition, strain-level variation and differences in experimental design – such as the timing of *A. muciniphila* administration relative to disease induction or therapy may markedly influence outcomes. Systematically addressing these sources of heterogeneity will be essential to reconcile conflicting findings and rationally design *A. muciniphila*-based interventions.

## The role of *A. muciniphila* in metabolic disorders

**Figure 1:**
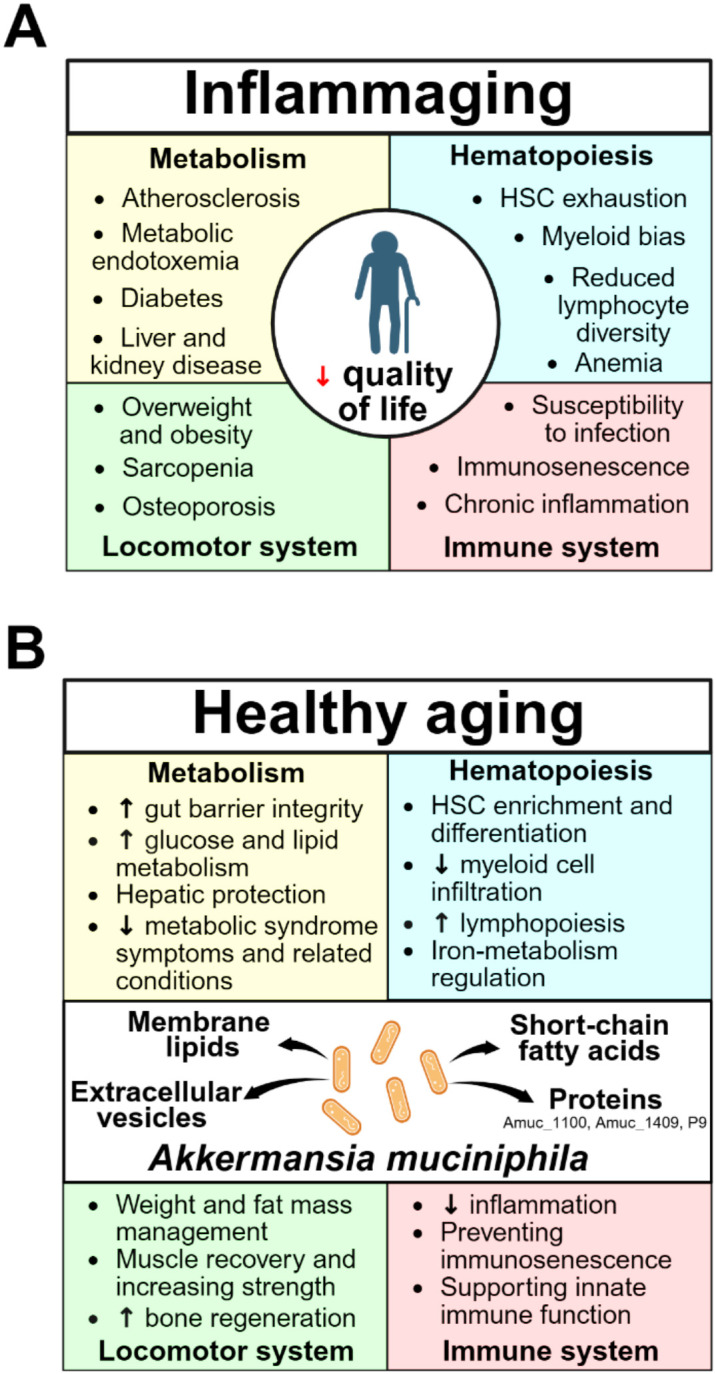
Potential beneficial effects of *Akkermansia muciniphila* towards anti-aging and alleviating age-associated disorders via microbial multiple different effector molecules. **(A)** Inflammaging, marked by the emergence of age-associated diseases and dysfunction of metabolism, hematopoiesis, immune, and locomotor systems, is a key factor in reducing the quality of life of older individuals. **(B)***A. muciniphila* promotes healthy aging by producing effector molecules that enhance overall physiological functions. By regulating mucin degradation and cross-feeding with other commensals, *A. muciniphila* promotes the generation of short-chain fatty acids (acetate, propionate and indirectly butyrate), which signal to host tissues to improve insulin sensitivity, lipid handling, and energy expenditure. In addition, *A. muciniphila* releases extracellular vesicles and expresses immunomodulatory outer membrane proteins such as Amuc_1100, which interact with host pattern-recognition receptors to modulate gut barrier function, GLP-1 secretion, and inflammatory signaling. Together, these metabolite- and vesicle-mediated pathways contribute to reduced adipose tissue inflammation, amelioration of hepatic steatosis, preservation of skeletal muscle function, modulation of bone and mineral metabolism, and support of neurocognitive resilience in aging, collectively enhancing metabolic health and systemic homeostatic capacity in older hosts.

Throughout aging the body undergoes complex metabolic alterations marked by disrupted glucose and lipid homeostasis, readily detectable in the blood (29). Elevated fasting glucose and progressive insulin resistance predispose older individuals to type 2 diabetes mellitus (**Figure 1A**). Concurrently, hepatic and adipose tissue dysfunction increases lipid fractions associated with accelerated aging (30). Such metabolic dysregulation promotes secondary complications, particularly cardiovascular diseases, which remain a major cause of mortality. Altered lipid metabolism, reflected in elevated cholesterol and triglyceride levels (31), fosters atherosclerotic plaque formation (32), while enhanced monocyte recruitment further increases vascular stiffness and disease progression (33, 34). Older adults also exhibit biochemical markers of organ decline, including reduced serum albumin and total protein (35) and elevated creatinine and urea levels indicative of hepatic or renal dysfunction (36). These disturbances contribute to sarcopenia, a progressive loss of muscle mass and strength, thereby reducing physical performance and quality of life (37). Given these widespread effects, restoring metabolic homeostasis during aging remains a critical therapeutic priority.

*Akkermansia muciniphila* is recognized for its capacity to modulate and improve systemic metabolic functions (**Figure 1B**). Its activity promotes the production of short-chain fatty acids (SCFAs), including acetate, butyrate, and propionate, whose levels decline with age (38–40). Beyond serving as substrates for gluconeogenesis and thus contributing to glucose homeostasis, these fatty acids interact with free fatty acid receptors (FFARs) in hepatocytes and adipocytes, enhancing lipid metabolism, stimulating energy expenditure, and inducing the secretion of anorexigenic peptides that suppress appetite (41–44). In addition, *A. muciniphila* secretes bioactive proteins that influence host metabolism. Notably, the P9 protein stimulates the release of glucagon-like peptide-1 (GLP-1) and restores thermogenic capacity in mice fed a high-fat diet (HFD) (45). Furthermore, administration of *A. muciniphila* or its outer membrane protein Amuc_1100 was shown to activate lipolysis, reduce adipose mass (46), and alleviate insulin resistance in experimental murine models (47).

A growing body of evidence indicates a positive correlation between *Akkermansia muciniphila* abundance and metabolic health in aging (**Table 1**). In murine models of obesity and diabetes, administration of *A. muciniphila* improved metabolic parameters, including glucose tolerance and lipid profiles (49, 68). Its anti-diabetic effects, mediated by extracellular vesicles, proteins, and metabolites, were confirmed in HFD-fed mice, where normalization of glucose homeostasis was observed (50, 51). Similarly, metformin-induced enrichment of *A. muciniphila* under HFD conditions improved glycemic control (69). The bacterium also exerts hypolipidemic effects: in fatty liver disease models, *A. muciniphila* and its components reduced body weight and plasma cholesterol and triglyceride levels (52, 53), while in ApoE^−/−^ mice, treatment decreased atherosclerotic plaque formation and circulating IL-6, cholesterol, and triglycerides (70). Pilot human studies corroborate these findings - pasteurized *A. muciniphila* improved insulin sensitivity, lowered cholesterol, and reduced body weight in overweight or type 2 diabetic individuals (48, 71). Moreover, *A. muciniphila* supplementation restored muscle function by upregulating *Igf1* expression in aging mice (72) and enhanced muscle strength in older adults (73). The distinct outcomes reported for viable versus pasteurized *A. muciniphila* likely reflect fundamentally different modes of action (28). Live bacteria can colonize, degrade mucin, and continuously reshape the intestinal ecosystem through metabolite production and niche competition. In contrast, pasteurized bacteria lack metabolic activity but retain surface and cell-wall components that signal through pattern-recognition receptors to modulate immunity and barrier function, potentially offering metabolic benefits with a lower risk of mucus erosion.

**Table 1:** System-level influence of *Akkermansia muciniphila* in various models of metabolic disorders.

№	Experimental system and disease model	Form, dose and introduction protocol of bacteria or antigen	Effect	Reference
1	Individuals with excess body weight, insulin resistance and metabolic syndrome.	Viable or pasteurized *A. muciniphila*, 10^10^ CFU, pes os for 3 months	↑ insulin sensitivity, ↓ body weight and fat mass, Blood serum: ↓ insulin level, ↓ total cholesterol, ↓ GGT, ↓ AST, ↓ LDH, ↓ CK	(48)
2	C57BL/6J mice, HFD	Viable *A. muciniphila*, 10^9^ CFU, oral gavage for 14 weeks; Recombinant protein P9 из *A. muciniphila*, 100 μg, pes os or intraperitoneal for 4 weeks	↓ body weight, ↑ glucose tolerance, ↑ thermogenesis, Blood serum: ↑ insulin level, ↑GLP-1	(45)
3	C57BL/6J mice, HFD	Viable or pasteurized *A. muciniphila*, 1,5*10^8^ CFU, oral gavage for 4–5 weeks; Recombinant protein Amuc_1100, 3 μg, oral gavage for 5 weeks	↓ body weight and fat mass, ↑ insulin sensitivity, ↑ glucose tolerance, ↓ triglycerides in serum	(47)
4	C57BL/6J mice, HFD	Viable or pasteurized *A. muciniphila*, 2*10^8^ CFU, oral gavage for 5 weeks;	↓ body weight and fat mass, ↑ energy expenditure, ↓ total cholesterol	(49)
5	C57BL/6J mice, HFD	Pasteurized *A. muciniphila*, 2*10^8^ CFU, oral gavage for 12 weeks;	↓ body weight gain, ↓ fat mass, ↑ glucose tolerance, Blood serum: ↓ fasting insulin level, ↓ fasting glucose level	(50)
6	C57BL/6J mice witn intestinal *Ffar4* deficiency	Pasteurized *A. muciniphila*, 5*10^10^ CFU, oral gavage for 6 weeks;	↑ glucose tolerance, Blood serum: ↑ insulin level, ↑GLP-1	(51)
7	C57BL/6 mice, HFD	Viable *A. muciniphila*, 10^9^ CFU, oral gavage for 5 weeks; OMVs from *A. muciniphila*, 10 μg, oral gavage 5 weeks	↓ body weight and fat mass, Blood serum: ↓ glucose level, ↓ total cholesterol, ↓ triglycerides	(52)
8	C57BL/6N, HFD	Viable *A. muciniphila*, 10^8–^10^9^ CFU, oral gavage for 10 weeks	No changes in body weight gain, Blood serum: ↓ triglycerides ↓ ALT, ↓ AST	(53)
9	C57BL/6 mice, HFD	Viable *A. muciniphila*, 1,5*10^9^ CFU, oral gavage for 21 weeks	↑glucose tolerance, ↓ body weight gain, ↓ fat mass, ↓ triglycerides in liver, Blood serum: ↓ ALT, ↓ AST	(54)
10	C57BL/6 mice, HFCD	Viable *A. muciniphila*, 10^8^ CFU, oral gavage for 6 weeks	↓ body weight and fat mass, ↓ triglycerides in liver, Blood serum: ↓ total cholesterol, ↓ ALT, ↓ AST, ↓ ALP, ↓ CK	(55)
11	E3L.CETP mice, western‐type diet	Viable *A. muciniphila*, 2*10^8^ CFU, oral gavage for 4 weeks	↓ body weight, Blood serum: ↓ total cholesterol, ↓ triglycerides	(56)
12	C57BL/6J mice, acute hyperlipidemia; CREBH-null (KO) mice, chronic hyperlipidemia	Viable *A. muciniphila*, 2*10^8^ CFU, oral gavage for 2 weeks	↑ clearance of triglycerides during acute hyperlipidemia, ↓ triglycerides, ↑glucose tolerance	(57)
13	*Apoe*^−/−^ mice on a C57BL/6 background, western‐type diet	Viable or pasteurized *A. muciniphila*, 5*10^9^ CFU, oral gavage for 9 weeks	↓ atherosclerotic lesion	(58)
14	C57BL/6 mice, HFD→ diabetes induction	OMVs from *A. muciniphila*, 10 μg, oral gavage 2 weeks	↓ body weight gain, ↑ glucose tolerance	(59)
15	C57BL/6 mice, CCl_4_-induced liver injury→HFD	Viable or pasteurized *A. muciniphila*, 10^9^ CFU, 4 weeks; OMVs from *A. muciniphila*, 50 μg, oral gavage 4 weeks	Blood serum: ↓ glucose level, ↓ total cholesterol, LDL, VLDL and HDL, ↓ triglycerides, ↓ ALT, ↓ AST	(60)
16	C57BL/6 mice, NIAAA	Viable *A. muciniphila*, 2*10^8^ CFU, oral gavage for 15 days	↓ triglycerides in liver, Blood serum: ↓ ALT, ↓ AST	(61)
17	C57BL/6J mice, HFD	Viable or pasteurized *A. muciniphila*, 2*10^8^ CFU, oral gavage for 4 weeks;	↓ body weight and fat mass, ↑ insulin sensitivity, ↓ fasting glucose level,	(62)
18	C57BL/6 mice	Viable or pasteurized *A. muciniphila*, 10^9^ CFU, oral gavage for 5 weeks;	↓ body weight and fat mass, Blood serum: ↓ glucose level, ↓ triglycerides, ↓ total cholesterol, ↓ AST	(63)
19	C57BL/6 mice	Viable *A. muciniphila* ATCC BAA-835, 2*10^8^ CFU, oral gavage for 5 weeks;	↓ body weight gain, ↓ fat mass, ↑ lean mass, ↑ insulin sensitivity, ↑ glucose tolerance, ↓ lipid accumulation in liver	(64)
20	C57BL/6 mice, HFD	Viable *A. muciniphila*, 10^8^ CFU, oral gavage for 12 weeks;	*↓* body weight gain, ↓ fat mass, ↑ glucose tolerance, ↓ lipid accumulation in liver Blood serum: ↓ triglycerides, ↓ insulin level	(65)
21	C57BL/6J mice, HFD; Beagles, HFD	Mice: pasteurized *A. muciniphila*, 2*10^8^ CFU Dogs: pasteurized *A. muciniphila*, 2*10^9^ CFU, oral gavage for 9 weeks	Mice: ↓ body weight and fat mass, ↓ fasting glucose level, ↓total cholesterol и LDL, Dogs: ↓ body weight, ↑ insulin sensitivity ↓ fasting glucose level, ↓total cholesterol, LDL and HDL, ↓ ALT	(66)
22	Dogs, HFD	Pasteurized *A. muciniphila* EB-AMDK19, 10^12^ CFU, oral gavage for 12 weeks;	↓ body weight gain, ↓ fat mass, ↓ triglycerides in serum	(67)

ALT, alanine aminotransferase; AST, aspartate aminotransferase; ALP, alkaline phosphatase; GGT, gamma-glutamyl transferase; CK, creatine kinase; GLP-1, glucagon-like peptide-1; LDL, low-density lipoprotein; VLDL, very-low-density lipoprotein; HDL, high-density lipoprotein; HFD, high-fat diet; HFCD, high-fat and cholesterol diet; NIAAA, chronic and binge ethanol feeding; OMVs, outer membrane vesicles.

Finally, aging also affects the inorganic blood components, leading to electrolyte imbalances. Among the most frequent are hypo- and hypernatremia, which commonly arise in the context of renal and cardiovascular dysfunction (74). Age-related alterations additionally disrupt calcium and phosphate metabolism due to impaired intestinal absorption, reduced levels of regulatory vitamins and hormones, and declining kidney function. Calcium imbalance contributes to bone fragility, manifesting as osteopenia and osteoporosis (**Figure 1A**), while hyperphosphatemia accelerates vascular aging and promotes vascular calcification, thereby increasing the risk of chronic cardiovascular disease (75). Interestingly, dietary calcium and phosphate intake can modulate gut microbiota composition, with low dietary levels favoring the proliferation of *Akkermansia muciniphila* (76). Conversely, the intestinal microbiome itself influences electrolyte balance and related physiological processes (77, 78). For instance, *A. muciniphila* was shown to affect intracellular calcium dynamics, suggesting that its molecular factors can modulate calcium signaling pathways (79). Moreover, oral administration of *A. muciniphila* promotes bone repair and regeneration by stimulating osteogenic activity and suppressing osteoclast-mediated bone resorption, a characteristic of aging (80, 81).

## *A. muciniphila* as a modulator of age-related hematopoietic decline

Aging is accompanied by a profound remodeling of the immune system, particularly hematopoiesis (82). Immunosenescence encompasses systemic alterations in innate and adaptive immunity, marked by chronic inflammation with elevated IL-6 and TNF, increased susceptibility to infections, and impaired tissue regeneration, all contributing to age-associated diseases (**Figure 1A**). These changes stem largely from hematopoietic stem cell (HSC) dysfunction and clonal restriction, characterized by reduced self-renewal and differentiation potential despite an overall increase in HSC number (83). Loss of repopulating capacity skews differentiation toward the myeloid lineage and suppresses lymphopoiesis, driven by both intrinsic alterations and extrinsic metabolic and endocrine cues from the bone marrow niche (84). Diminished lymphoid hematopoiesis along with thymic involution results in fewer naïve B and T cells and an accumulation of plasma cell clones and memory T cells (85), while enhanced myelopoiesis increases circulating proinflammatory monocytes (86, 87). Finally, aging is associated with the mobilization of atypical, hyperactivated neutrophils, whose excessive activation through NETosis or degranulation can aggravate comorbidities such as stroke or infection (88).

Although hematopoiesis is primarily regulated through epigenetic and transcriptional mechanisms, as well as by growth factors and cytokines within the bone marrow microenvironment, external influences such as the intestinal microbiota also play a significant role (89, 90). Several studies demonstrated that reduced microbial diversity leads to a decline in bone marrow HSC numbers, while dysbiosis in aged mice drives a shift in HSC differentiation toward myelopoiesis through IL-1R-dependent signaling (91). As a key constituent of a healthy gut microbiota, *Akkermansia muciniphila* can modulate immune regulation and hematopoietic remodeling both directly and indirectly (**Table 2**). By preserving intestinal barrier integrity, *A. muciniphila* limits translocation of bacterial components into circulation, thereby mitigating IL-1R-mediated alterations in HSC differentiation (102). Conversely, excessive proliferation of *A. muciniphila*, which disrupts the mucin layer, has been associated with leukocytosis characterized by increased neutrophil and monocyte proportions and a concomitant reduction in lymphocytes (27).

**Table 2:** System-level influence of *Akkermansia muciniphila* in various models of immune and hematopoietic impairment.

№	Experimental system and disease model	Form, dose and introduction protocol of bacteria or antigen	Effect	Reference
Hematopoiesis				
1	C57BL/6 mice	Viable *A. muciniphila* ATCC BAA-835, 10^7–^10^9^ CFU, i.v. for2 weeks; Membrane fraction *A. muciniphila*, 10^8^ CFU, i.v. for 2 weeks	Rapid activation BM myelopoiesis, slow and long-lasting hepato-splenomegaly and extramedullary hematopoiesis, mobilization, expansion, and differentiation of HSPCs in spleen, long‐lasting anemia	(92)
2	C57BL/6J mice, CCl_4_-induced liver injury	Viable *A. muciniphila* DSM 22959, 10^9^ CFU, oral gavage for 6 weeks; Cell-free supernatant from *A. muciniphila*, 200 μl, 6 weeks	Modulation of the hepcidin–ferroportin axis, which regulates systemic iron metabolism, thereby ensuring iron availability for erythropoiesis	(93)
Adaptive immunity				
3	C57BL/6J mice, DSS-induced colitis	Viable *A. muciniphila*, 1,5*10^9^ CFU, oral gavage for 3 weeks	↓ intestinal inflammation and disease severity, induction and expansion of suppressive RORγt+ Treg, promotion of immune tolerance	(94)
4	C57BL/6 mice	Viable *A. muciniphila*, 10^9^ CFU, oral gavage for 5 weeks	Stimulation of lymphopoiesis: induction of T_FH_ cell responses, differentiation and activation of *A. muciniphila* –specific T cells and B-cells, ↑ production of *A. muciniphila* –specific IgG1.	(95)
Innate immunity				
5	*In vitro*: BMDM from C57BL/6 mice and human monocytes	Activation with viable or pasteurized *A. muciniphila*, 10^6^ CFU	Long-term reprogramming of innate immune cells, promoting an anti-inflammatory phenotype	(96)
6	C57BL/6J mice, DSS-induced colitis	Viable or pasteurized *A. muciniphila* ATCC BAA-835, 3*10^9^ CFU, oral gavage for 2 weeks	↓ intestinal inflammation and colonic damage, ↓ colonic monocyte and neutrophil populations, stimulation of IL-22 secretion, expansion of retinoic-acid–producing CD103^+^ dendritic cells	(97)
7	C57BL/6J mice, aging	Viable *A. muciniphila*, 2*10^8^ CFU, oral gavage for 4 weeks;	↓ systemic inflammation, ↑ innate immune functions: chemotaxis, phagocytosis, NK cells activity, ↓ oxidative stress parameters in peritoneal leukocytes	(98)
8	C57BL/6 mice, LPS-induced acute lung injury	Viable *A. muciniphila* ATCC BAA-835, 3*10^9^ CFU, oral gavage for 4 weeks	↓ pulmonary inflammatory response, ↓ macrophage and neutrophil infiltration	(99)
9	C57BL/6 mice, HDM- induced allergic asthma	Pasteurized *A. muciniphila* EB-AMDK19, 2,5*10^8^ CFU, 18 days	↓ airway hyper-responsiveness and inflammation, ↓ eosinophil infiltration, suppression of Th2 responses	(100)
10	C57BL/6J mice, periodontitis	Viable or pasteurized *A. muciniphila* ATCC BAA-835, 10^9^ CFU, oral gavage for 6 weeks; Recombinant protein Amuc_1100, 6 μg, oral gavage for 6 weeks	↑ presence of M2 macrophages, ↑ expression of *Il10* anti-inflammatory response	(101)

HSPCs, hematopoietic stem and progenitor cells.

The interaction between *Akkermansia muciniphila* and the immune system is pivotal for maintaining peripheral immune tolerance. Through the production of immunomodulatory metabolites and the presentation of pathogen-associated molecular patterns (PAMPs), *A. muciniphila* contributes to the regulation of host immune responses to commensal microbiota. For instance, bacterial diacylphosphatidylethanolamine was identified as a key molecule mediating immunomodulatory effects via activation of TLR2 on immune cells (103). Moreover, *A. muciniphila* exhibits immunosuppressive activity, reducing inflammatory cell infiltration in models of colitis (104). Several studies demonstrated that *A. muciniphila* and its components promote macrophage polarization toward the anti-inflammatory M2 phenotype leading to increased production of IL-10 (105), decreased production of TNF (105) and an overall attenuation of inflammation. Additionally, enrichment with *A. muciniphila* has been associated with an increased abundance of RORγt^+^ regulatory T cells in the intestine (94) and enhanced secretion of IL-22 by ILC3s, driven by retinoic acid-dependent signaling from dendritic cells (97). Collectively, these findings indicate that *A. muciniphila* promotes immune homeostasis by shifting the intestinal environment toward a tolerogenic state.

Recent work also highlights the influence of *A. muciniphila* on hematopoiesis (**Figure 1B**). Oral delivery of the bacterium or its outer membrane vesicles (OMVs) activated myelopoiesis and induced extramedullary hematopoiesis, accompanied by splenomegaly and hepatomegaly through TLR- and IL-1R-dependent mechanisms (92). Although this represents a stress-induced rather than homeostatic response, it underscores the importance of IL-1R signaling in microbiota-driven hematopoietic regulation. In addition, *A. muciniphila*-derived vesicles can circulate systemically, enhancing intestinal barrier integrity and alleviating DSS-induced colitis (106, 107). Notably, the bacterium itself can translocate beyond the gut to the bloodstream and bone marrow (108), where it may contribute to immune tolerance via Treg expansion and exert long-term modulatory effects on hematopoiesis during aging.

Erythropoiesis also undergoes significant alterations with aging. Anemia, characterized by reduced hematocrit and hemoglobin levels, is particularly prevalent among the elderly. As myelopoiesis increases, erythroid differentiation becomes less efficient, leading to a decline in reticulocyte numbers. This anemia is multifactorial, arising from age-related disruptions in the bone marrow niche and systemic imbalances in hormones, vitamins, and iron metabolism aggravated by chronic inflammation and comorbid diseases (109–111). *Akkermansia muciniphila* may influence erythropoietic activity through its impact on iron metabolism. The gut microbiota as a whole contributes to local iron availability for HSC renewal and erythroid differentiation by regulating hemoglobin processing in bone marrow macrophages (112). Specifically, *A. muciniphila* and its components modulate hepatic hepcidin expression in models of CCl_4_-induced fibrosis (93) and in activated macrophages (113). In these settings, hepcidin exerts a protective function, limiting fibrotic progression while serving as a key regulator of systemic iron homeostasis and bioavailability, suggesting that *A. muciniphila* may indirectly support erythropoietic balance during aging.

*Akkermansia muciniphila* has been shown to exert systemic anti-inflammatory and immunomodulatory effects relevant to aging (**Figure 1B**). Administration of the bacterium reduced chronic inflammation, notably IL-6 production, in both peripheral blood and the hippocampus, thereby improving cognitive function in aged mice (114). In a murine model of osteoporosis, one month of *A. muciniphila* supplementation enhanced innate and adaptive immunity, increasing chemotaxis, phagocytosis, NK cell activity, and lymphocyte proliferation (98). Moreover, oral administration extended lifespan in mice, reinforcing its protective role in aging (115).

## Conclusion

Taken together, the accumulated evidence positions *Akkermansia muciniphila* as one of the most compelling microbial candidates for combating the physiological aging-associated decline (**Figure 1**). Through its integrated and pleiotropic actions - reinforcing intestinal barrier integrity, suppressing local and systemic inflammation, optimizing metabolic and immune homeostasis, and modulating hematopoietic balance - A*. muciniphila* demonstrates the capacity to counteract multiple hallmarks of aging and sustain organismal resilience (**Tables 1**, **2**).

Despite its remarkable therapeutic potential, key questions remain unresolved regarding the long-term safety, dose dependence, and context-specific efficacy of *A. muciniphila*-based interventions (116). Dose-dependent and even opposing outcomes observed across models highlight the need for rigorous, individualized approaches to its clinical application (28). In particular, the molecular and cellular mechanisms through which *A. muciniphila* regulates hematopoietic stem cell function, lineage commitment, and bone marrow niche homeostasis warrant systematic investigation. Moreover, deeper insights are required into how *A. muciniphila* integrates into the complex microbial ecosystem of the elderly gut and how its colonization reshapes the abundance and activity of other taxa implicated in healthy longevity. The roles of specific *A. muciniphila*–derived metabolites and postbiotic molecules in modulating cellular senescence, genomic stability, and DNA repair remain an especially promising but underexplored frontier.

Future research should shift from correlative observations to mechanistic elucidation and translational development. It will be critical to establish standardized, safe, and effective formulations using live, pasteurized, or postbiotic derivatives, to advance *A. muciniphila* from a biomarker of healthy aging to a bona fide therapeutic tool. Ultimately, harnessing this unique symbiont offers a powerful and biologically grounded avenue for developing personalized, microbiota-based geroprotective strategies aimed at extending the health span and improving quality of life in the aging population.
